# Enhanced Chemoprevention of Prostate Cancer by Combining Arctigenin with Green Tea and Quercetin in Prostate-Specific Phosphatase and Tensin Homolog Knockout Mice

**DOI:** 10.3390/biom14010105

**Published:** 2024-01-14

**Authors:** Qiongyu Hao, Susanne M. Henning, Clara E. Magyar, Jonathan Said, Jin Zhong, Matthew B. Rettig, Jaydutt V. Vadgama, Piwen Wang

**Affiliations:** 1 Division of Cancer Research and Training, Charles R. Drew University of Medicine and Science, Los Angeles, CA 90059, USA; qiongyuhao@cdrewu.edu (Q.H.); jayvadgama@cdrewu.edu (J.V.V.); 2 David Geffen School of Medicine, University of California, Los Angeles, CA 90095, USA; 3 Center for Human Nutrition, David Geffen School of Medicine, University of California, Los Angeles, CA 90095, USA; 4 Department of Pathology, David Geffen School of Medicine, University of California, Los Angeles, CA 90095, USA; 5 VA Greater Los Angeles Healthcare System, Los Angeles, CA 90073, USA; 6 Department of Internal Medicine, School of Medicine, University of California, Riverside, CA 92521, USA; 7 Departments of Medicine and Urology, David Geffen School of Medicine, University of California, Los Angeles, CA 90095, USA

**Keywords:** green tea, quercetin, arctigenin, prostate cancer, chemoprevention, PTEN knockout mouse, combination

## Abstract

The low bioavailability of most phytochemicals limits their anticancer effects in humans. The present study was designed to test whether combining arctigenin (Arc), a lignan mainly from the seed of *Arctium lappa*, with green tea (GT) and quercetin (Q) enhances the chemopreventive effect on prostate cancer. We performed in vitro proliferation studies on different cell lines. We observed a strong synergistic anti-proliferative effect of GT+Q+Arc in exposing androgen-sensitive human prostate cancer LNCaP cells. The pre-malignant WPE1-NA22 cell line was more sensitive to this combination. No cytotoxicity was observed in normal prostate epithelial PrEC cells. For an in vivo study, 3-week-old, prostate-specific PTEN (phosphatase and tensin homolog) knockout mice were treated with GT+Q, Arc, GT+Q+Arc, or the control daily until 16 weeks of age. In vivo imaging using prostate-specific membrane antigen (PSMA) probes demonstrated that the prostate tumorigenesis was significantly inhibited by 40% (GT+Q), 60% (Arc at 30 mg/kg bw), and 90% (GT+Q+Arc) compared to the control. A pathological examination showed that all control mice developed invasive prostate adenocarcinoma. In contrast, the primary lesion in the GT+Q and Arc alone groups was high-grade prostatic intraepithelial neoplasia (PIN), with low-grade PIN in the GT+Q+Arc group. The combined effect of GT+Q+Arc was associated with an increased inhibition of the androgen receptor, the PI3K/Akt pathway, Ki67 expression, and angiogenesis. This study demonstrates that combining Arc with GT and Q was highly effective in prostate cancer chemoprevention. These results warrant clinical trials to confirm the efficacy of this combination in humans.

## 1. Introduction

Prostate cancer is the most commonly diagnosed male malignancy and the second-leading cause of cancer death among men in the United States [1]. Most prostate tumors are slow-growing, with a period of about 20–25 years from initiation to the stage when the clinically detectable phenotype can be identified. Many patients die with prostate cancer without any symptoms [2]. However, aggressive prostate cancer cases metastasize rapidly, particularly to the bones and lymph nodes, often before symptoms are noticed [3]. It remains a challenge to distinguish aggressive tumors from indolent ones. Patients may be over-treated with conventional methods such as surgery and androgen deprivation therapy, suffering side effects from these treatments, including incontinence, impotence, and osteoporosis [4,5]. Since prostate cancer is typically diagnosed in the elderly population and its growth and progression rate is relatively slow, it may be a good candidate disease for chemoprevention [6]. Chemoprevention is defined as the use of agents to suppress, retard, or prevent either the initial stage of carcinogenesis or the progression of premalignant cells to invasive disease [7,8]. Even a slight delay in disease progression may result in substantial reduction in the incidence of clinically detectable prostate cancer [6].

**Figure 1:**
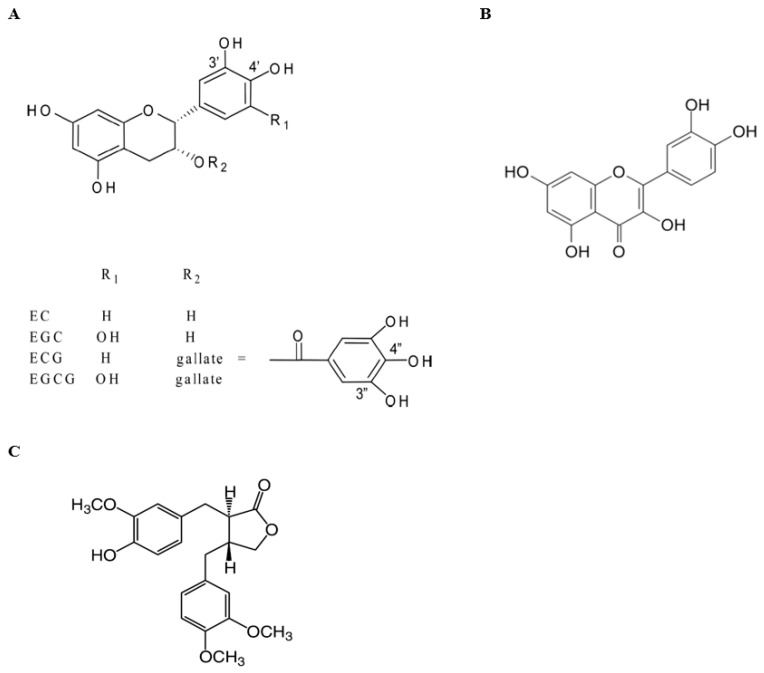
Chemical structures. (**A**) Green tea polyphenols; (**B**) quercetin; (**C**) arctigenin.

Natural products, particularly phytochemicals from the diet and plants, have become a significant resource for developing less-/non-toxic chemopreventive agents. However, the low bioavailability of most phytochemicals, like green tea polyphenols (GTPs, chemical structures in Figure 1A), and their extensive metabolism in vivo limit their anticancer efficacy in humans. Effective doses, as demonstrated in in vitro studies, are challenging to achieve in vivo through oral consumption and at safe levels [9,10]. Green tea (GT) is produced from the leaves of the plant *Camellia sinensis*, with epigallocatechin-3-gallate (EGCG) as the most abundant and bioactive component [11]. In our prior work, we were able to demonstrate that the combination of quercetin (Q, chemical structure in Figure 1B) with GT significantly increases tissue concentrations of GTPs and decreases the methylation of GTPs into less-active metabolites, leading to an enhanced inhibition of prostate tumor growth in mice, associated with an increased inhibition of several critical signaling pathways involved in prostate cancer, including the androgen receptor (AR) and PI3K/Akt pathways [12]. Q is a flavonoid in most edible vegetables and fruits, mainly onions, apples, and red wine. It is a natural inhibitor of both multidrug resistance protein 1 (MRP1), a significant exporter in the regulation of GTPs’ cellular absorption, and catechol-*O*-methyltransferase (COMT), which catalyzes the methylation of GTPs [13,14,15,16]. However, Q has poor water solubility and a low bioavailability. Efforts directed toward increasing the bioavailability/bioactivity of Q include the development of water-soluble forms of Q [17], the use of nanoparticle delivery systems [18], and combinations with other phytochemicals [12,19].

To further optimize the combination of GT and Q, we conducted an in vitro screening of additional phytochemicals. We found that arctigenin (Arc, chemical structure in Figure 1C) most potently synergized with both GT and Q. Arc is a lignan found mainly in the seeds of the herb Arctium lappa. It also exists in other plants, including *Bardanae fructus*, *Saussurea medusa*, and *T. nucifera*. The anticancer activity of Arc has been observed in several types of cancer, including pancreatic [20,21], breast [22,23], and lung [24] cancer. We demonstrated in in vitro and animal models that Arc is a potent inhibitor of prostate tumor growth in non-obese and obese cases, partly through its inhibition of the AR and PI3K/Akt pathways [25,26].

In the present study, we tested the combined effect of Arc with GT and Q in vitro and an autochthonous mouse model of prostate cancer in prostate-specific PTEN (phosphatase and tensin homolog) knockout (KO) mice. PTEN is a tumor suppressor gene mutated frequently in many primary human cancers [27]. PTEN encodes a phosphatase that recognizes lipid and peptide substrates, including PI3K. Through PI3K, PTEN controls Akt signaling and its downstream targets responsible for cell size, motility, cell cycle, and death [28]. Mutations in the PTEN catalytical domain lead to the loss of phosphatase and tumor suppressor activity. Prostate-specific PTEN KO mice develop murine prostatic intraepithelial neoplasia (mPIN) at 6 weeks, invasive adenocarcinoma at 9 weeks, and metastasis to the lymph nodes and lungs after 12 weeks of age, which recapitulates the disease progression seen in humans [29].

## 2. Materials and Methods

### 2.1. Cell Line and Cell Culture

The androgen-sensitive LNCaP human prostate cancer cell line was purchased from ATCC (Chicago, IL, USA). The LNCaP cells were maintained in an RPMI 1640 medium, supplemented with 10% (*v*:*v*) fetal bovine serum, 100 IU/mL of penicillin, and 100 µg/mL of streptomycin at 37 °C in a 5% CO_2_ incubator. The WPE1-NA22 cell line was derived from a non-tumorigenic human prostate epithelial cell line, RWPE-1, after exposure to a chemical carcinogen, N-methyl-N-nitrosourea, and selected and cloned in vivo and in vitro [30]. It mimics a pre-malignant stage of prostatic intra-epithelial neoplasia (PIN). The WPE1-NA22 cells were purchased from ATCC and cultured in a keratinocyte serum-free medium supplemented with 0.05 mg/mL of bovine pituitary extract and 5 ng/mL of epidermal growth factor (Invitrogen, Carlsbad, CA, USA). Normal prostate epithelial PrEC cells were purchased from Lonza Walkersville, Inc. (Walkersville, MD, USA) and cultured in a PrEGM medium (Lonza Walkersville, Inc.). All cell lines were tested periodically for mycoplasma contamination using a PCR-based Universal Mycoplasma Detection kit (ATCC).

### 2.2. Cell Proliferation Assay

The LNCaP and WPE1-NA22 cells were seeded into opaque-wall 96-well plates at a density of 8 × 10^3^ per well. An inhibition curve was achieved for individual compounds by incubating the cell lines with a series of concentrations of EGCG, Q, or Arc (Sigma-Aldrich, St. Louis, MO, USA) for 48 h. The doses that led to about a 20–30% cell growth inhibition with each compound were selected for the combination study. Cell proliferation was measured with an adenosine triphosphate (ATP) assay using the CellTiter-Glo^®^ Luminescent cell viability assay kit (Promega Corporation, Madison, WI, USA). To minimize the effect of hydrogen peroxide that may be formed via the autoxidation and/or dimerization of phytochemicals in the cell culture medium [31], 50 units/mL of catalase was added to the medium prior to EGCG, Arc, and Q. The experiment was repeated twice, with four wells for each treatment in each experiment.

A combination index (CI) was calculated for the combinations of EGCG, Q, and Arc using the CompuSyn software (version 1.0, ComboSyn, Inc., Paramus, NJ, USA), which is based on the widely-accepted Chou–Talalay equation and mass-action law [32]. A value of CI less than 1 indicates a synergistic effect of a combination; equal to 1 is additive, and greater than 1 is antagonistic [32].

### 2.3. Preparation of GT, Q Diet, and Arc Solution for Animal Study

GT was freshly prepared thrice a week on Monday, Wednesday, and Friday by brewing one tea bag (Celestial Seasonings, Boulder, CO, USA) in 240 mL of boiling water for 5 min. The composition of GTPs in the brewed tea was (mg/L) EGCG 388 ± 12, EGC 204 ± 4, EC 44 ± 2, ECG 64 ± 7, and catechin 7 ± 1. The GT was administered as drinking water ad libitum. Q (Sigma-Aldrich, St Louis, MO, USA) was supplemented into the AIN-93G diet at a concentration of 0.2%, as customized by Dyets Inc. (Bethlehem, PA, USA). Arc (ausmausco pharma Co., Ltd., Basel, Switzerland) was dissolved in a vehicle (2% DMSO in corn oil) and administered via oral gavage at 30 mg/kg of body weight daily.

### 2.4. Animal Study

All procedures carried out using mice were approved by the Institutional Animal Care and Use Committee at Charles R. Drew University of Medicine and Science. Prostate-specific PTEN KO mice have a homozygous PTEN knockout in the prostate, and they were generated by crossing ARR2Probasin-Cre transgenic mice (PB-Cre4) with PTEN^LoxP/LoxP^ mice (The Jackson Laboratory, Sacramento, CA, USA). PB-Cre4 mice carry the Cre gene under the control of a derivative of the rat prostate-specific probasin (PB) promoter—ARR2PB. An RT-PCR detection of Cre mRNA in PB-Cre 4 mice demonstrated that Cre expression is post-natal and prostate-specific [33]. The male offspring with the PTEN^loxP/−^ × PB-Cre4 genotype were crossed with PTEN^LoxP/LoxP^ females. Only F2 generations of male offspring with the PTEN^loxP/loxP^ × PB-Cre4 (PTEN KO) genotype were used for this study [29]. The mice were genotyped using a standard PCR with 3 primers for PTEN (5′-TCCCAGAGTTCATACCAGGA-3′, 5′-GCAATGGCCAGTACTAGTGAAC-3′, and 5′-AATCTGTGCATGAAGGGAAC-3′ [34]) and 2 primers for Cre (5′- CAAAACAGGTAGTTATTCGG-3′ and 5′-CGTATAGCCGAAATTGCCAG-3′ [35]). The prostate-specific knockout of PTEN was further confirmed via a Western blot analysis of the PTEN protein expression in different tissues, including the liver, lung, kidney, and prostate, where the prostate was the only tissue without PTEN expression (Supplementary Figure S1).

A total of 40 male PTEN KO mice were weaned at 3 weeks of age and randomly assigned to 4 groups, receiving GT+Q, Arc, GT+Q+Arc, or the control. Their food and water consumption was measured 3 times per week, and the mouse body weight was measured once per week. Tumor growth/metastasis was monitored every 3 weeks using a Pearl^®^ Trilogy Small Animal Imaging System (LI-COR Biotechnology, Lincoln, NE, USA) after an i.v. injection of IRDye 800 CW YC-27, a near-infrared dye-labeled optical probe specifically designed to target prostate-specific membrane antigens (PSMAs). PSMAs are a unique membrane-bound glycoprotein that is over-expressed in prostate cancer, including metastatic disease [36]. The mice were sacrificed at 16 weeks of age, and their prostates/tumors, livers, lungs, and blood were collected. 

### 2.5. Pathological Evaluation of Tumor Stage

At the time of sacrifice, the lower urogenital tract (bladder, seminal vesicles, and prostate) was removed en bloc and further microdissected under a dissecting microscope. A section of the right dorsolateral and anterior prostate with seminal vesicles was fixed in 10% phosphate-buffered formalin, paraffin-embedded, and sectioned for histology. The remaining tissues were snap-frozen in liquid nitrogen and stored in a −80 °C freezer until use. H&E stained slides were assessed for their tumor stage under a microscope by two pathologists independently, using the Bar Harbor Classification system according to the consensus report of the Mouse Model of Human Cancer Consortium Prostate Pathology Committee [37]. A Gleason score was assigned to the primary lesions of each prostate based on the degree of differentiation. The lungs and livers were also paraffin-embedded and H&E stained for the examination of metastasis.

### 2.6. Western Blot Analysis

For the in vitro study, LNCaP cells were treated with EGCG (20 µM) + Q (2.5 µM), Arc (1 µM), EGCG+Q+Arc, or the control; WPE1-NA22 cells were treated with EGCG (20 µM) + Q (2.5 µM), Arc (0.5 µM), EGCG+Q+Arc, or the control, for 24 h. The cells were harvested and total protein was extracted using an RIPA lysis buffer (Santa Cruz, CA, USA). For the animal study, total protein was extracted from the prostate/tumor tissues using the RIPA lysis buffer (Santa Cruz). The procedure for the Western blot analysis was described previously [38]. Briefly, 50 μg of protein was separated on a 4–12% Bis-Tris gel (Invitrogen), electrotransferred to nitrocellulose membranes, and blocked in 5% nonfat milk. The membranes were incubated with primary antibodies for the detection of AR (sc-7305, 1:500 dilution), PTEN (sc-7974, 1:500 dilution) (Santa Cruz Technology, Santa Cruz, CA, USA), Akt (4685, 1:1000 dilution), and p-Akt (4058, 1:1000 dilution) (Cell Signaling Technology, Danvers, MA, USA), respectively. GAPDH was used as a loading control.

### 2.7. Tissue Microarray and Immunohistochemical Analysis of Ki67 and Microvessel Density

A section of each prostate/tumor was fixed in 10% phosphate-buffered formalin and paraffin-embedded for a tissue microarray and immunohistochemical detection as described previously [12]. The tissue array was assembled with 5 cylindrical cores from each donor block. The slides were incubated with monoclonal anti-mouse Ki67 or anti-mouse CD31 antibody for the microvessel evaluation (DAKO North America Inc., Carpinteria, CA, USA) and counterstained with hematoxylin. The slides were scanned on a ScanScope AT (Aperio Technologies, Inc., Vista, CA, USA), and a morphometric analysis was performed digitally with Definiens’ Tissue Studio (Definiens Inc., Parsippany, NJ, USA) in a non-biased method at the UCLA Department of Pathology. Briefly, the nuclear detection module and classification tool were pre-defined to identify positive and negative nuclei within each core. Thresholds were set to classify negative nuclei with hematoxylin staining and positive nuclei with DAB staining. The data were exported to Excel for statistical analysis.

### 2.8. Statistical Analysis

The SPSS software (Version 24, Chicago, IL, USA) was applied for the statistical analysis. The mean values and standard deviation (SD) were calculated. A comparison of means was performed via a one-way analysis of variance (ANOVA) with Tukey’s post-test for paired comparisons or a mixed-effect model for longitudinal data of repeated measures. Differences were considered significant if *p* < 0.05.

## 3. Results

### 3.1. Synergistic Anti-Proliferative Effects In Vitro of the Combination of Arc with EGCG and Q

**Figure 2:**
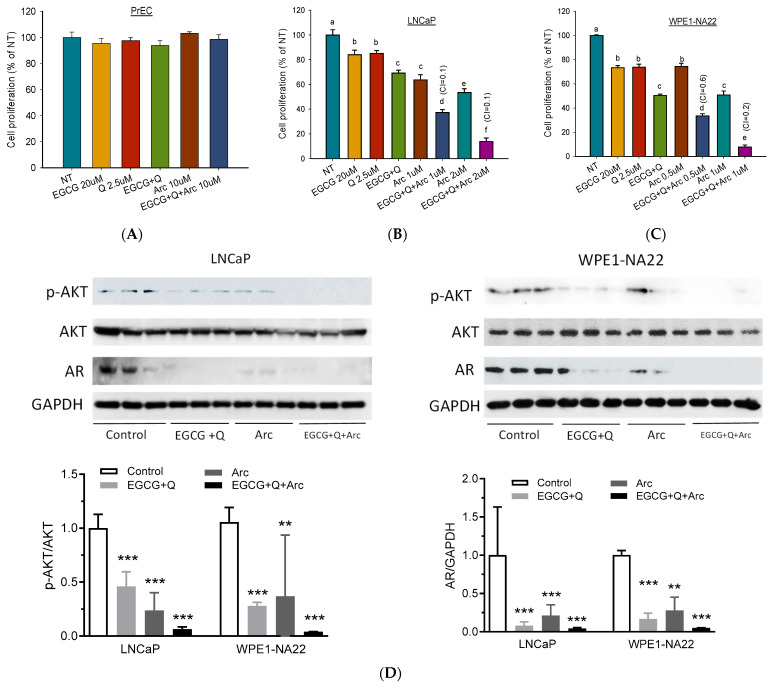
Arc with EGCG and Q synergistically increased the anti-proliferative effect in cultured prostate cancer cells. Androgen-sensitive human prostate cancer LNCaP cells (**A**), prostate pre-malignant WPE1-NA22 cells (**B**), and normal prostate epithelial PrEC cells (**C**) were treated with the indicated concentrations of EGCG, Q, Arc, or their combinations for 48 h. Cell proliferation was measured via an adenosine triphosphate (ATP) assay. Data are presented as mean ± SD. EGCG, Q, and Arc combined effect was calculated with a combination index (CI). A value of CI less than 1 indicates a synergistic effect, equal to 1 is additive, and greater than 1 is antagonistic. To analyze the modulation of protein markers, LNCaP and WPE1-NA22 cells were treated with control, EGCG at 20 µM + Q at 2.5 µM, Arc at 1 µM (LNCaP) or 0.5 µM (WPE1-NA22), or EGCG+Q+Arc for 24 h. Total protein was extracted for Western blot analysis of AR, Akt, and p-Akt levels (**D**). NT, non-treatment, DMSO control; EGCG, epigallocatechin-3-gallate; Q, quercetin; Arc, arctigenin; AR, androgen receptor. Columns with different letters indicate a significant difference between treatments, *p* < 0.05. ** *p* < 0.01, and *** *p* < 0.001 compared to control. Original images can be found in Supplementary File S1.

To investigate the chemopreventive effect of EGCG+Q+Arc in vitro, we selected the early-stage androgen-sensitive human prostate cancer LNCaP cells and the pre-malignant WPE1-NA22 cells. When used individually, Arc demonstrated the highest potency in the inhibition of LNCaP and WPE1-NA22 cell proliferation compared to EGCG or Q (Figure 2A). The combination of EGCG with Q significantly increased the anti-proliferative effect compared to EGCG or Q alone. In contrast, adding Arc further increased the effect of EGCG + Q by 2–5-fold in an Arc-dose-dependent manner. The combination of EGCG+Q +Arc achieved a strong synergistic effect, as indicated by a CI value of 0.1 (Figure 2A). The pre-malignant WPE1-NA22 cells were more sensitive to the three compounds. The combination of EGCG + Q with Arc at lower doses nearly eliminated the WPE1-NA22 cells (Figure 2B). In contrast, no cytotoxicity was observed in normal prostate epithelial PrEC cells with Arc up to 10 µM, either alone or in combination with EGCG and Q (Figure 2C). The Western blot analysis showed that both EGCG+Q and Arc were able to inhibit the proliferation-related signaling molecules, including AR expression and Akt phosphorylation, with an increasing strength with the combination of EGCG+Q+Arc (Figure 2D). These results indicate a significantly enhanced anti-proliferative effect in combining Arc with EGCG+Q compared to EGCG+Q or Arc alone, and the combined effect was selective to cancer cells without affecting normal cells.

### 3.2. Increased Inhibition of Prostate Tumorigenesis by Arc in Combination with GT and Q

**Figure 3:**
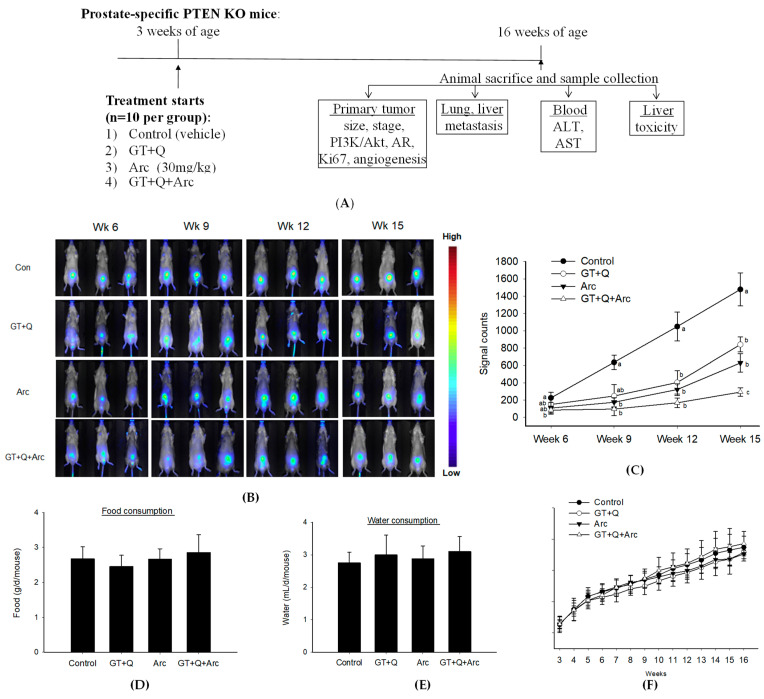
Enhanced prostate tumorigenesis inhibition by combining GT, Q, and Arc. Prostate-specific PTEN knockout mice (3 weeks old, *n* = 10 per group) were administered with GT+Q (GT as drinking water + 0.2% Q in diet), Arc (30 mg/kg of body weight daily via oral gavage), GT+Q+Arc, or control until 16 weeks of age. Tumor development was monitored through in vivo imaging every 3 weeks. (**A**) Study flow chart. Representative images are shown in (**B**) and results in (**C**). Food (**D**) and water (**E**) consumption was measured 3 times a week, and mouse body weight (**F**) once a week. Data are presented as mean ± SD. Con, control; GT, green tea; Q, quercetin, Arc: arctigenin. Different letters at each time point indicate a significant difference between groups, *p* < 0.05.

A flow chart of the chemoprevention study in PTEN KO mice is presented in Figure 3A. Treatments started at 3 weeks of age when the mouse prostate was in a pre-neoplastic state. In vivo imaging demonstrated that both GT+Q and Arc alone significantly inhibited the formation of prostate tumors in the PTEN KO mice compared to the control. At the same time, the combination of Arc with GT and Q enhanced the inhibitory effect significantly compared to GT+Q or Arc alone (Figure 3B,C). At week 15, tumor formation was inhibited by 90% with the GT+Q+Arc treatment as compared to the control (Figure 3C). There was no difference in food (Figure 3D) or water (Figure 3E) consumption or in mouse body weight (Figure 3F) among groups during the intervention. There was no significant liver toxicity in any of the groups, as evaluated with liver pathological examinations and with blood alanine aminotransferase (ALT) and aspartate aminotransferase (AST) levels. These in vivo outcomes confirmed the increased chemopreventive effect of GT+Q+Arc compared to GT+Q or Arc alone, as well as the safety of this combination.

### 3.3. Amelioration of the Prostate Pathology by GT, Q, and Arc

**Figure 4:**
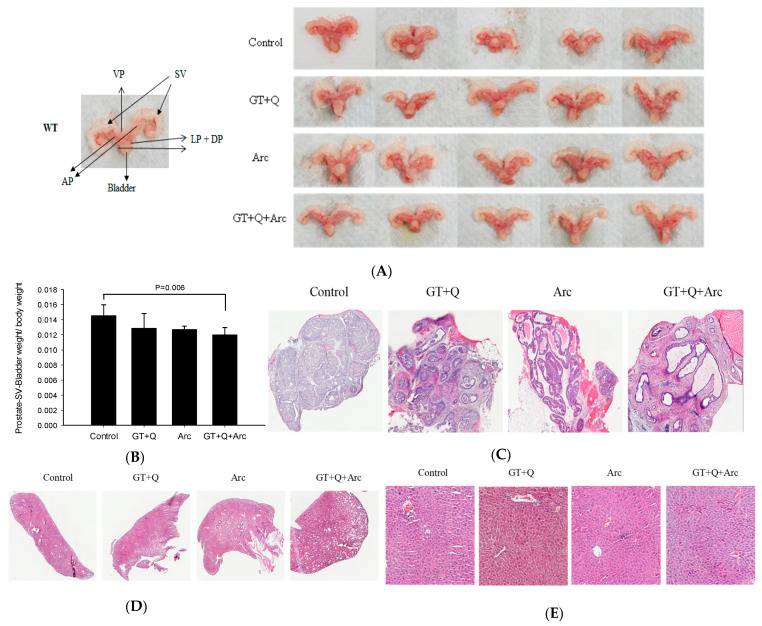
Pathological analysis of the mouse prostate (tumor) tissues. The mouse lower urogenital tract (bladder, seminal vesicles, and prostate) was removed en bloc at the time of sacrifice. Representative images of the prostates/tumors are shown in (**A**), with enlarged and hardened prostates in the control group and normal prostates in the GT+Q+Arc group. The data of the lower urogenital tract weight is shown in (**B**). Representative images of H&E-stained prostates/tumors for tumor stage evaluation are shown in (**C**), with invasive adenocarcinoma in the control group, high-grade PIN in the GT+Q and Arc groups, and low-grade PIN in the GT+Q+Arc group. No metastasis was observed in the lung (**D**) or liver (**E**). Con, control; GT, green tea; Q, quercetin, Arc: arctigenin. Data are presented as mean ± SD.

All control mice had hardened and enlarged prostates at the time of sacrifice, while the mouse prostates in the GT+Q+Arc group appeared normal (Figure 4A,B). The weight of the lower urogenital tract (bladder, seminal vesicles, and prostate) was slightly (but not significantly) reduced by both the GT+Q and Arc treatments compared to the control. In contrast, a significant reduction in the weight of the lower urogenital tract was achieved with the GT+Q+Arc treatment (Figure 4B). A pathological examination showed that all control mice had an invasive adenocarcinoma (Gleason score 8–10) as the primary lesion in the prostate. The primary lesion in the GT+Q and Arc groups was high-grade PIN (Gleason score 5–7), while in the GT+Q+Arc group, the dominant lesion was low-grade PIN (Gleason score 2–4) (Figure 4C). No metastasis was observed in the lung or liver in any of the groups (Figure 4D,E). These pathologic results corroborated the observations of significantly enhanced anti-tumorigenic strength by combining Arc with GT and Q.

### 3.4. Modulation of Signaling Molecules Involved in Proliferation and Angiogenesis

**Figure 5:**
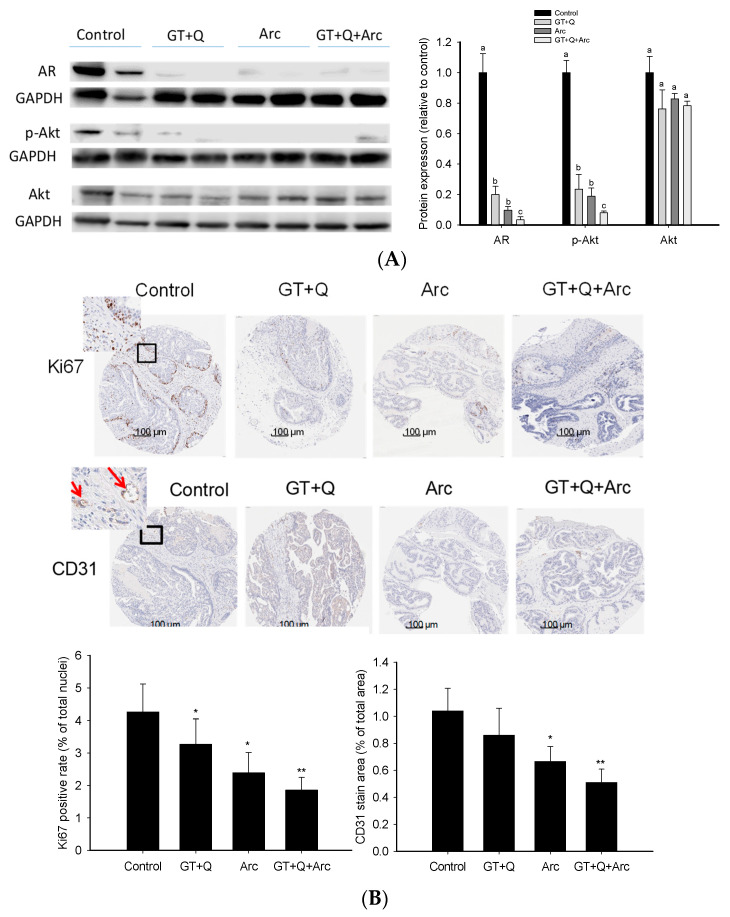
Modulation of signaling pathways/events by the combination treatment. The expression/phosphorylation of signaling molecules involved in the AR and PI3K/Akt pathways in the prostate/tumor tissues were analyzed with the Western blot method and are quantitated in (**A**). A prostate/tumor tissue section was formalin-fixed and paraffin-embedded for tissue microarray and immunohistochemical detection. Slides were cut and incubated with monoclonal antibodies for Ki67 and CD31 (for microvessel staining). Slides were counterstained with hematoxylin. Nuclei were stained in blue and Ki67 or CD31 in brown (**B**). The red arrows indicate microvessels (**B**). The positive rates of Ki67 nuclear staining and microvessel density are presented as mean ± SD (**B**). Con, control; GT, green tea; Q, quercetin, Arc: arctigenin. Different letters in each protein category indicate a significant difference between groups, *p* < 0.05. Compared to the control, * *p* < 0.05, ** *p* < 0.01. Original images can be found in Supplementary File S1.

The Western blot analysis demonstrated the strong ability of these phytochemicals to regulate both the AR and PI3K/Akt signaling pathways. GT+Q and Arc alone were able to reduce the AR protein expression as well as Akt phosphorylation significantly. At the same time, the most potent effect was achieved with GT, Q, and Arc in combination (Figure 5A). Likewise, the IHC analysis of the tumor tissue microarray showed that Ki67 expression in the tumors was significantly reduced by GT+Q or Arc alone. The strongest effect was achieved with the combination of GT, Q, and Arc (Figure 5B). Also, treatment with Arc alone significantly decreased tumor angiogenesis compared to the control, as evaluated via the CD31 staining of microvessels. The combination of Arc with GT and Q further increased the anti-angiogenic effect (Figure 5B). These results showed that the combined effect of GT+Q+Arc was at least partially through an enhanced modulation of multiple signaling pathways/events.

## 4. Discussion

These results support the notion that the combinatorial use of phytochemicals is a promising means to enhance their chemopreventive effect at lower doses of individual compounds. Continuous efforts have been directed to overcoming the limitations of the low bioavailability of phytochemicals like GT and Q, and some encouraging results have been seen with the development of more water-soluble derivatives and formulation with nanoparticle delivery systems [17,39,40,41,42]. For instance, compared to classical dihydroquercetin (also called taxifolin, TAX), a water-soluble form of TAX (aqTAX) showed more potent antihypoxic activities against cerebral ischemia. Therefore lower doses of aqTAX may be needed in the clinical treatment of cerebral ischemia, thereby preventing the toxic side effects of TAX [41]. The combinatorial use of phytochemicals may provide a non-/less-toxic manner in which to additively/synergistically enhance the anticancer strength of individual compounds. In the present study, Arc in combination with GT and Q demonstrated high effectiveness in the inhibition of prostate tumorigenesis, with a significantly increased strength compared to GT+Q or Arc alone both in vitro and in PTEN KO mice. Previously, we found that Arc was 10–20-fold more potent than GTPs in inhibiting prostate cancer cell growth, and its tumor bioavailability was about 50-fold higher than that of GTPs [25,26]. We also demonstrated in vitro that Arc synergistically enhanced the anti-proliferative effect of both GT and Q in prostate and breast cancer cells [19,43]. Furthermore, the safety of the combination of GT+Q+Arc at their adequate dose levels was confirmed in the present study. Together, these results predict the success of GT+Q+Arc to be a highly effective regimen in the chemoprevention of prostate cancer clinically. This combination may also be used during active surveillance in early-stage prostate cancer patients to suppress disease progression [44].

Treatment with the GT+Q+Arc combination was associated with an enhanced modulation of multiple signaling pathways/events involved in carcinogenesis. GT+Q and Arc can inhibit the PI3K/Akt and AR signaling pathways, as previously found by our group and other investigators [12,19,25,26,45]. At the same time, the combination of Arc with GT+Q further increased the inhibitory effect on these pathways, as shown in the present study. The PI3K/Akt and AR pathways are critical modulators of the development and progression of prostate cancer by mediating the transcription of target genes that regulate the growth and differentiation of prostate epithelial cells [46]. Increasing evidence indicates that the PI3K/Akt/mTOR pathway may directly regulate the expression and activation of AR [46], which necessitates a dual inhibition of both the AR and PI3K/Akt pathways for a durable control of prostate cancer. In addition, the combination of GT+Q+Arc increasingly inhibited tumor angiogenesis compared to GT+Q or Arc alone, as indicated by microvessel density in mouse prostates/tumors in the present study. Angiogenesis manifests in the formation of new blood vessels from pre-existing ones, which is an essential step for solid tumor growth, and is therefore a potential target in cancer chemoprevention and treatment [47,48]. Together, the multi-targeting activity of GT, Q, and Arc is deemed to be an advantageous and important feature in the control of cancer, considering that a cancer may have hundreds of gene mutations/dysfunctions and many signaling pathways that crosstalk with each other [49,50]. By targeting multiple pathways/events involved in cancer development and progression, it may prevent or delay the incidence of chemoresistance and obtain a sustainable chemopreventive/therapeutic effect.

## 5. Summary and Conclusions

In summary, this study demonstrates that combining Arc with GT and Q synergistically increased the anti-proliferative effect in vitro, both in early-stage prostate cancer LNCaP cells and in pre-malignant WPE1-NA22 cells, without affecting normal prostate epithelial cells. Our in vivo study confirmed the enhanced chemopreventive effect of this combination in prostate-specific PTEN KO mice, as indicated by an increased inhibition of prostate carcinogenesis and Ki67 expression by GT+Q+Arc compared to the control, GT+Q, or Arc alone. The combined effect of GT+Q+Arc was associated with an increased modulation of multiple critical signaling pathways/events involved in prostate cancer carcinogenesis, including the AR and PI3K/Akt pathways and angiogenesis. These results warrant future clinical trial studies to translate this highly promising regimen into human applications, starting with dose-escalation studies to determine the recommended doses of GT, Q, and Arc in combination with phase II studies. Considering the non-toxic nature of these phytochemicals and their combinations, this study is anticipated to bring near-term benefits to populations at high risk for prostate cancer and patients with early-stage prostate cancer.

## Data Availability

The datasets supporting the conclusions of this article are included within the article.

## Supplementary Materials

The Supplementary figures and original images for Western blot results can be downloaded at: https://www.mdpi.com/article/10.3390/biom14010105/s1.

## Abbreviations

AR	androgen receptor
Arc	arctigenin
ATP	adenosine triphosphate
CI	combination index
COMT	catechol-O-methyltransferase
EC	epicatechin
ECG	epicatechin-3-gallate
EGC	epigallocatechin
EGCG	epigallocatechin-3-gallate
GT	green tea
GTPs	green tea polyphenols
KO	knockout
MRP	multidrug resistance-associated protein
mTOR	mammalian target of rapamycin
NT	non-treatment
PI3K	phosphatidylinositol 3-kinases
PIN	prostatic intraepithelial neoplasia
PSMA	prostate-specific membrane antigen
PTEN	phosphatase and tensin homolog
Q	quercetin
SCID	severe combined immunodeficiency
