# Correction: Hao et al. Enhanced Chemoprevention of Prostate Cancer by Combining Arctigenin with Green Tea and Quercetin in Prostate-Specific Phosphatase and Tensin Homolog Knockout Mice. *Biomolecules* 2024, *14*, 105

**DOI:** 10.3390/biom15020160

**Published:** 2025-01-21

**Authors:** Qiongyu Hao, Susanne M. Henning, Clara E. Magyar, Jonathan Said, Jin Zhong, Matthew B. Rettig, Jaydutt V. Vadgama, Piwen Wang

**Affiliations:** 1 Division of Cancer Research and Training, Charles R. Drew University of Medicine and Science, Los Angeles, CA 90059, USA; qiongyuhao@cdrewu.edu (Q.H.); jayvadgama@cdrewu.edu (J.V.V.); 2 David Geffen School of Medicine, University of California, Los Angeles, CA 90095, USA; 3 Center for Human Nutrition, David Geffen School of Medicine, University of California, Los Angeles, CA 90095, USA; shenning@mednet.ucla.edu; 4 Department of Pathology, David Geffen School of Medicine, University of California, Los Angeles, CA 90095, USA; cmagyar@mednet.ucla.edu (C.E.M.); jsaid@mednet.ucla.edu (J.S.); 5 VA Greater Los Angeles Healthcare System, Los Angeles, CA 90073, USA; jin.zhong@va.gov; 6 Department of Internal Medicine, School of Medicine, University of California, Riverside, CA 92521, USA; 7 Departments of Medicine and Urology, David Geffen School of Medicine, University of California, Los Angeles, CA 90095, USA; mrettig@mednet.ucla.edu

The authors would like to replace Figure 3B of the following published paper [1]. In the originally published Figure 3B, two mouse images in the GT+Q+Arc group were misplaced to incorrect time points within the GT+Q+Arc group. Also, a mouse image from the GT+Q group at Week 15 was placed twice at that time point. These mistakes have been corrected. The corrected Figure 3 is attached below:

The authors apologize for any inconvenience caused and would like to state that their scientific conclusions are unaffected. This correction was approved by the Academic Editor. The original publication has also been updated.

## Figures and Tables

**Figure 3:**
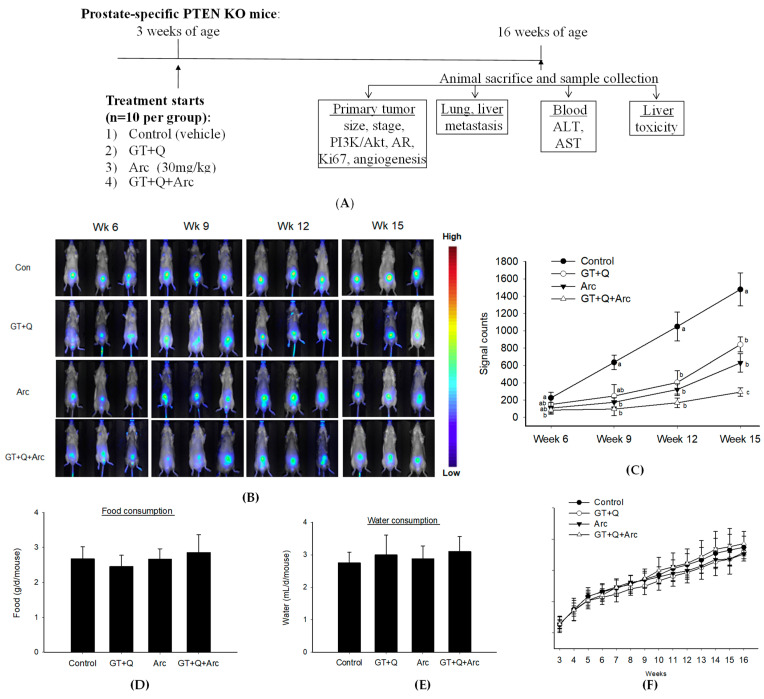
Enhanced prostate tumorigenesis inhibition by combining GT, Q, and Arc. Prostate-specific PTEN knockout mice (3 weeks old, *n* = 10 per group) were administered with GT+Q (GT as drinking water + 0.2% Q in diet), Arc (30 mg/kg of body weight daily via oral gavage), GT+Q+Arc, or control until 16 weeks of age. Tumor development was monitored through in vivo imaging every 3 weeks. (**A**) Study flow chart. Representative images are shown in (**B**) and results in (**C**). Food (**D**) and water (**E**) consumption was measured 3 times a week, and mouse body weight (**F**) once a week. Data are presented as mean ± SD. Con, control; GT, green tea; Q, quercetin, Arc: arctigenin. Different letters at each time point indicate a significant difference between groups, *p* < 0.05.
