# Supplementary material for: Enhanced Chemoprevention of Prostate Cancer by Combining Arctigenin with Green Tea and Quercetin in Prostate-Specific Phosphatase and Tensin Homolog Knockout Mice
Source: Biomolecules. 2024 Jan 14;14(1):105. doi: 10.3390/biom14010105 (PMC10813217; doi:10.3390/biom14010105)
Supplement: Supplementary file 1 [file biomolecules-14-00105-s001.zip › biomolecules-2814574-supplementary.pdf]

Supplementary Figures:

1.

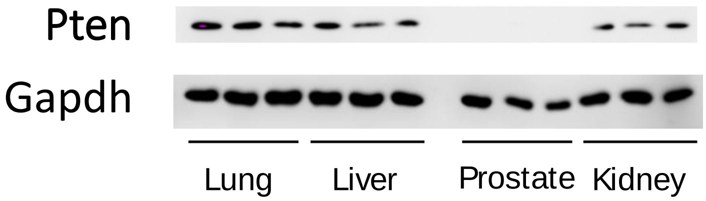

Figure S1. Western blot analysis of PTEN protein expression in prostate-specific PTEN knockout mice. Three mice were randomly selected and tissue samples, including lung, liver, prostate, and kidney from each of the mice analyzed for PTEN expression. PTEN was detected in the lung, liver, and kidney tissues, while not detectable in the prostate. Gapdh was used as loading control.

Original images for Western blot results:

1. **Original images for blots in Supplementary Figure S1:**

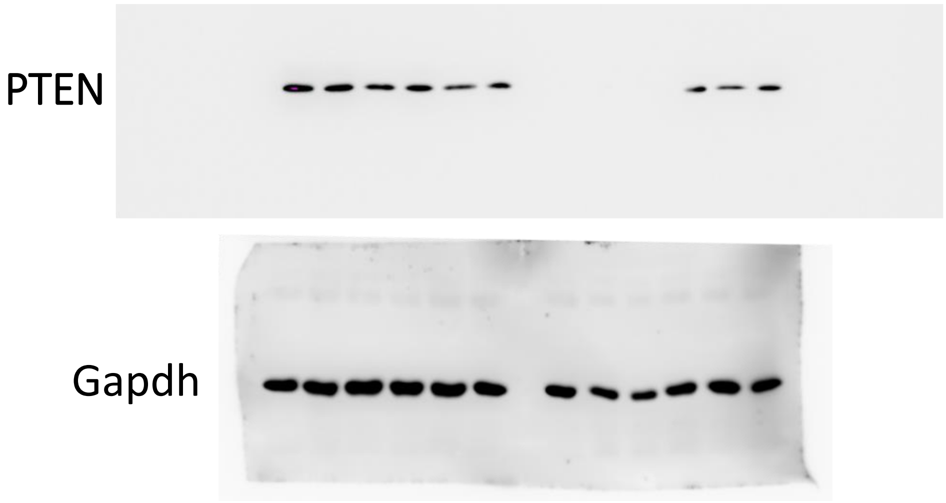

2. **Original Images for Blots in Figure 2.**

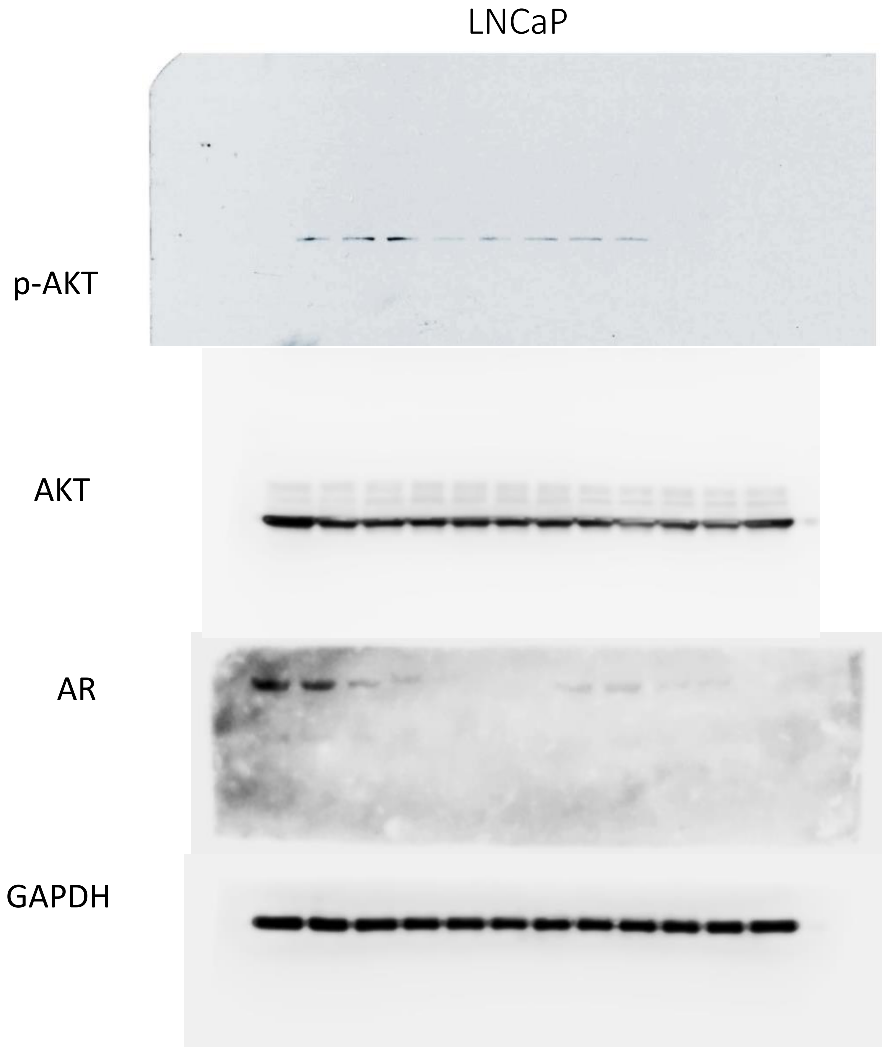

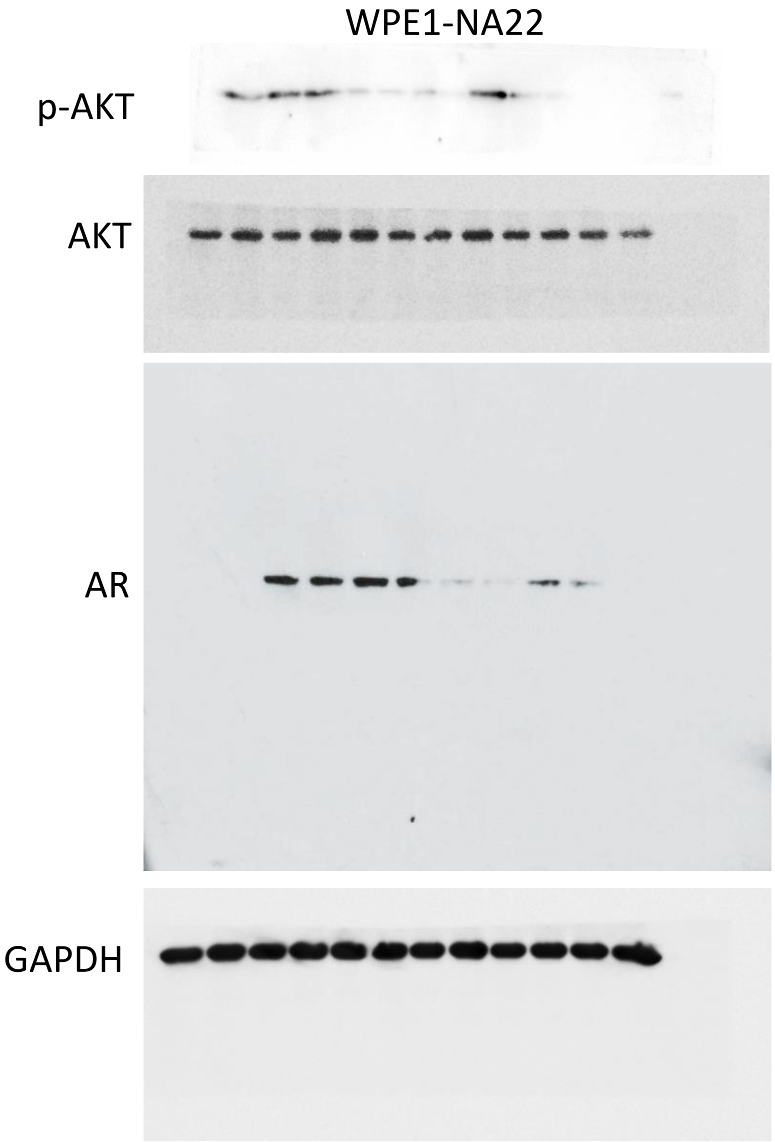
WPE1-NA22
p-AKT
AKT
AR
GAPDH

3. **Original Images for Blots in Figure 5.**

Gel-1

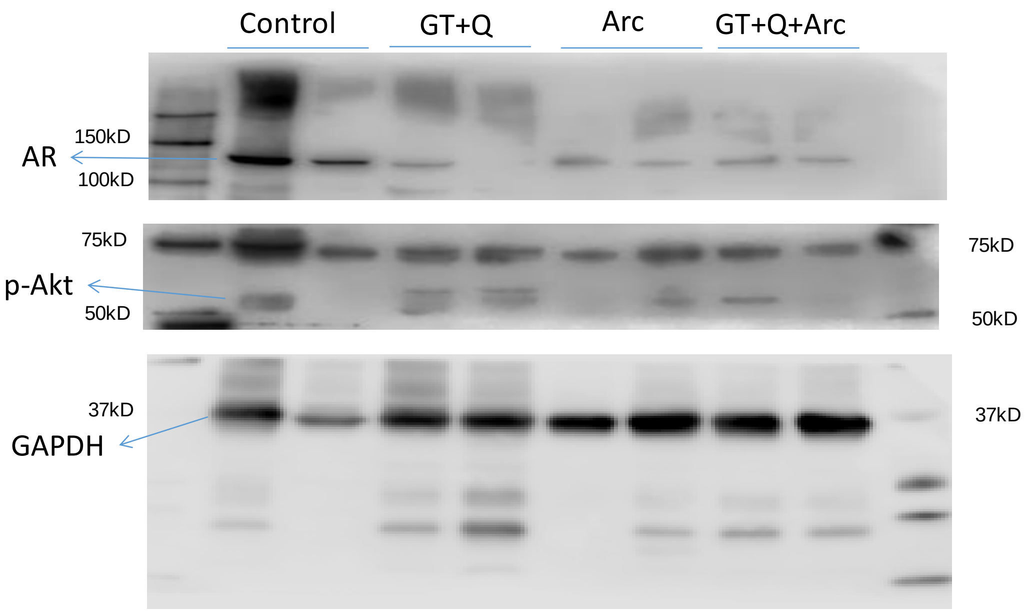

Gel-2

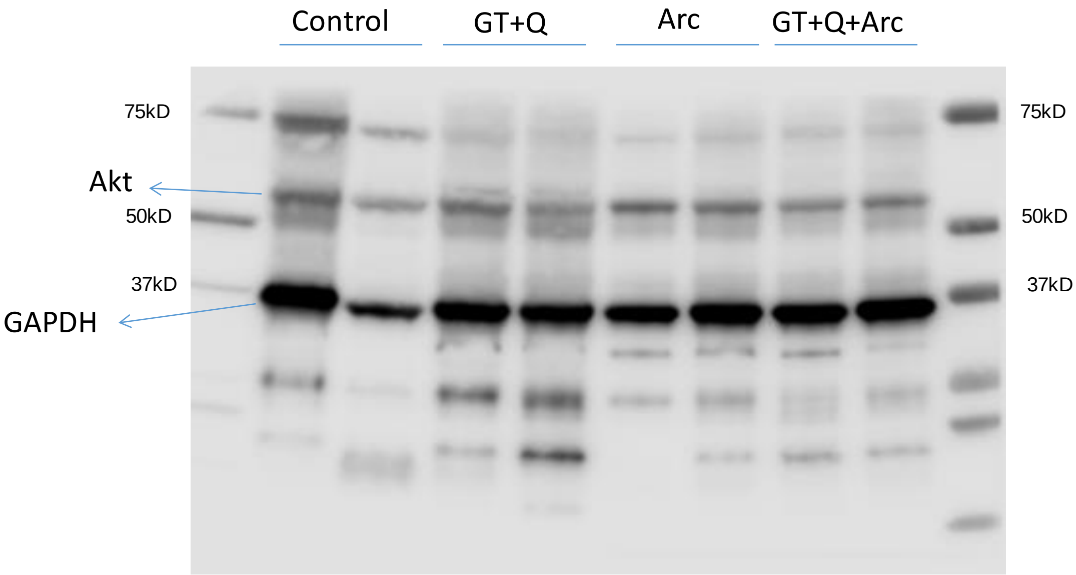

Gel-3

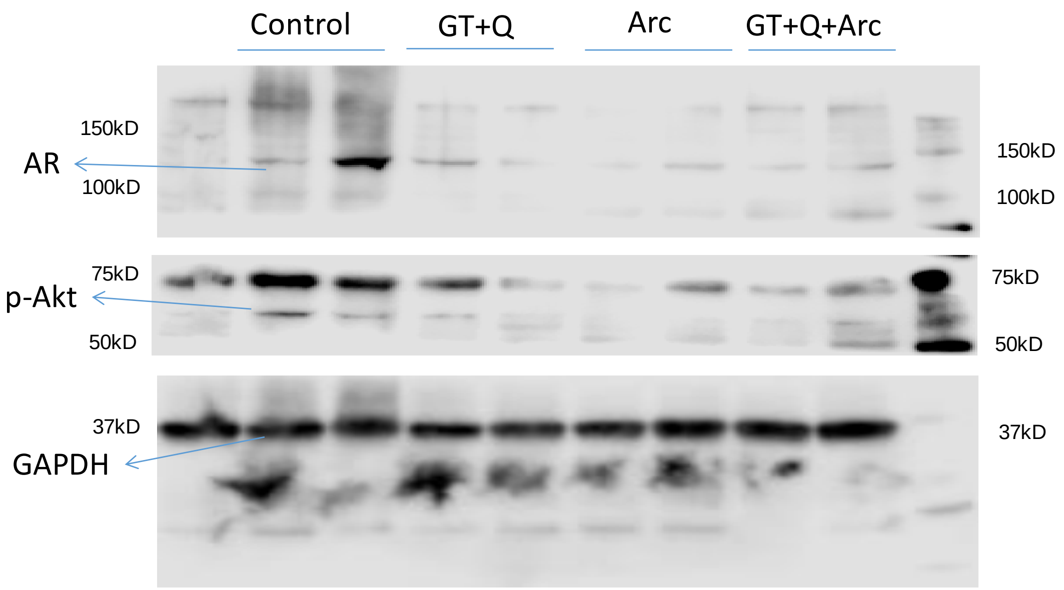
Control
GT+Q
Arc
GT+Q+Arc
150kD
AR
100kD
75kD
p-Akt
50kD
37kD
GAPDH
150kD
100kD
75kD
50kD
37kD

Gel-4

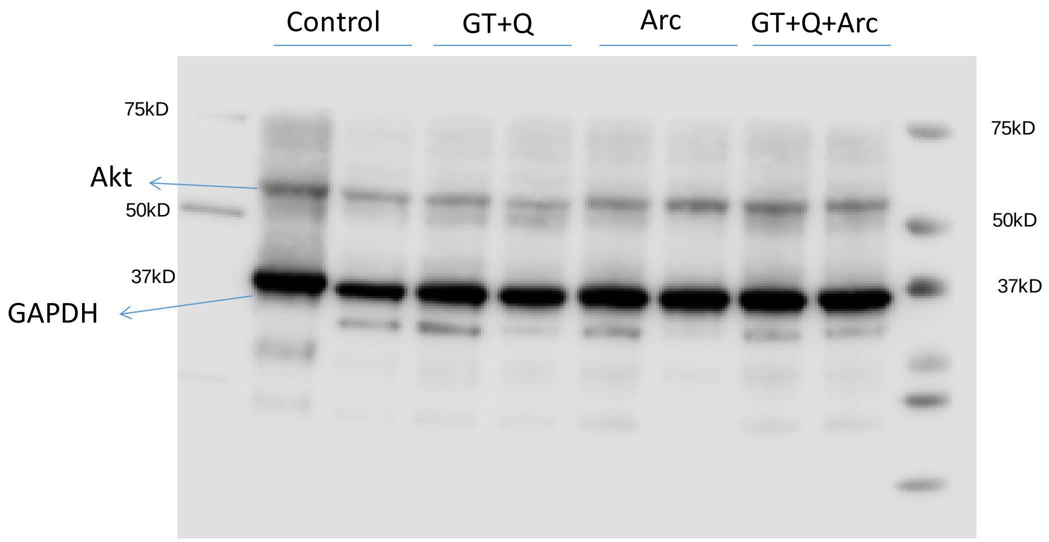
Control
GT+Q
Arc
GT+Q+Arc
75kD
Akt
50kD
37kD
GAPDH
75kD
50kD
37kD
